# Erytrocyte membrane anionic charge in type 2 diabetic patients with retinopathy

**DOI:** 10.1186/1471-2415-4-14

**Published:** 2004-10-08

**Authors:** Yasemin Budak, Hakan Demirci, Muberra Akdogan, Dilek Yavuz

**Affiliations:** 1 SSK Sevket Yilmaz Hospital, Section of Clinical Chemistry, Turkey; 2 SSK Sevket Yilmaz Hospital, Section of Family Medicine, Turkey; 3 SSK Sevket Yilmaz Hospital, Section of Ophthalmology, Turkey; 4 Marmara University Medical School, Section of Endocrinology and Metabolism, Turkey

## Abstract

**Background:**

The Steno hypothesis states that changes in basement membrane anionic charge leads to diabetic microvascular complications. In diabetic nephropathy, loss of basement membrane glycosaminoglycans and the association between glomerular basement membrane heparan sulphate and proteinuria has been documented. A correlation between erythrocyte surface and the glomerular capillary wall charges has also been observed.

The aim of this study is to evaluate the relationship between retinopathy and erythrocyte anionic charge and urinary glycosaminoglycan excretion in type 2 diabetic patients.

**Methods:**

49 subjects (58 ± 7 yrs, M/F 27/22) with type 2 diabetes with proliferative retinopathy (n = 13), nonproliferative retinopathy (n = 13) and without retinopathy (n = 23) were included in the study. 38 healthy subjects were selected as control group (57 ± 5 yrs, M/F 19/19). Erythrocyte anionic charge (EAC) was determined by the binding of the cationic dye, alcian blue. Urinary glycosaminoglycan and microalbumin excretion were measured.

**Results:**

EAC was significantly decreased in diabetic patients with retinopathy (255 ± 30 ng alcian blue/10^6 ^RBC, 312 ± 30 ng alcian blue/10^6 ^RBC for diabetic and control groups respectively, p < 0.001). We did not observe an association between urinary GAG and microalbumin excretion and diabetic retinopathy. EAC is found to be negatively corralated with microalbuminuria in all groups.

**Conclusions:**

We conclude that type 2 diabetic patients with low erythrocyte anionic charge are associated with diabetic retinopathy. Reduction of negative charge of basement membranes may indicate general changes in microvasculature rather than retinopathy. More prospective and large studies needs to clarify the role of glycosaminoglycans on progression of retinopathy in type 2 diabetic patients.

## Background

Diabetic retinopathy is the leading cause of blindness in diabetic adults [1,2]. During the first two decades of the disease, nearly all patients with type 1 diabetes and > 60 % of type 2 diabetes have retinopathy. The duration of the diabetes is probably the strongest predictor for development and progression of retinopathy [3]. The incidence of retinopathy positively correlates with HbA_1c _[4].

Diabetic patients with evidence of nephropathy are characterized by a 5 to 10 times higher incidence of proliferative retinopathy [5]. Albuminuria not only is associated with kidney disease but, is also a strong predictor of cardiovascular disease and proliferative retinopathy, suggesting it reflects a generalized vascular disease [6]. Thus, the coincidence of generalized vascular dysfunction, albuminuria, mesangial expansion, proliferative retinopathy and accelerated development of atherosclerosis suggests a common cause of abnormalities in susceptible diabetic patients.

The Steno hypothesis proposed that increased loss of anionic charge from basement membranes leads to diabetic microangiopathy [7]. This hypothesis has been proven for diabetic nephropathy but it's not clear for diabetic retinopathy.

Anionic content of basement membranes predominantly produced by glycosaminoglycan molecules (GAG). They constitute the charge selective barrier of the glomerul basement membrane [8]. Several clinical studies indicate that loss of the GAG is associated with diabetic albuminuria in diabetic and experimental models [9,10].

Although abnormal GAG metabolism is a well known phenomen in diabetic nephropathy, it is still debated whether it has a pathogenic role in diabetic retinopathy. The aim of this study was to evaluate the relationship between diabetic retinopathy and erythrocyte anionic charge as well as the urinary glycosaminoglycan excretion in type 2 diabetic patients.

## Methods

49 outpatients (27 male, 22 female) with type 2 diabetes mellitus diagnosed after the age of 30, were included and divided into 3 subgroups according to severety of retinopathy. Diabetes was diagnosed according to American Diabetes Association criteria. The onset of diabetes was after the age of 30 in these patients.

The study was approved by the institutional ethics committee and patients gave written informed consent. Exclusion criteria included hypertension, myocardial infarction, cerebrovascular disease, heart failure, treatment with antiaggregants, steroids or other drugs that effect blood pressure and glucose, serum creatinine > 200 μmol/l, connective tissue disorders and other systemic disease.

Renal and bladder infection diseases of patients and controls were excluded biochemically and microbiologically.

The ocular fundus was examined by an ophthalmologist after dilation of the pupils and classified. The patients were divided into three groups according to the severity of retinopathy. Group 1 consisted of 23 patients without retinopathy (R_0_). Group 2 consisted of 13 patients with nonproliferative retinopathy (R_1_-hemorrhages, microaneurysms, cotton-wool exudates). The 13 patients in group 3 (R_2_) had proliferative retinopathy (R_2_-neovascularisations, vitreous hemorrhages, ablatio of the retina).

Age and sex matched 38 healthy volunteers were included as healthy controls. Demographic characteristics of the groups are shown in Table 1.

Venous samples were collected after an overnight fast and 24 hour urine samples collected.

The charge on erythrocytes (RBC) was measured by means of cationic dye alcian blue 8GX (Sigma catalogue no: A 5268) according to the method of Levin with minor modifications as follows: [11]. From citrated venous blood samples, platelets and leukocytes were removed using the method of Beutler et al. [12]. After removal of platelets and leucocytes erythrocytes were washed 3 times in phosphate buffered saline (PBS), and subsequently a fraction of RBC was resuspended in PBS containing alcian blue at a final concentration of 250 mg/l. After a 30 min incubation at 37°C, the RBC suspension was centrifuged, and the remaining alcian blue concentration was measured in the supernatant with a Shimadzu UV 1201 V spectrophotometer (Shimadzu, Japan) at a wavelength of 650 nm. Each determination represents the mean of 2 assays. The quantity of alcian blue bound to RBCs was expressed as nanograms of alcian blue per 10^6 ^RBCs. In our experimental conditions the intraassay and interassay coefficients of variation were 5.8 and 7.6 % for 100 nanograms of alcian blue per 10^6 ^RBCs.

Urinary glycosaminoglycan (U_GAG_) excretion was determined in 24 hr urine samples spectrophotometrically at 520 nm by the addition of dimethylmethylene blue (Aldrich Chem Co., USA) and bovine kidney heparan sulfate as standard (Sigma catalogue no H 7640) [13]. In our experimental conditions the intraassay and interassay coefficients of variation were 1.5 and 2.4 % for 10 mg/l glycosaminoglycan, respectively.

Serum sialic acid was measured in serum with a colorimetric method described by Svennerholm with a Shimadzu UV-1201 spectrophotometer (Shimadzu, Japan) with a wavelength of 525 nm. Using N-acetyl neuraminic acid (Sigma catalogue no: A 3007) as standard [14]. Intra assay and interassay coefficients of variations were 6.6 and 9.2 % for 1 mmol/l sialic acid respectively.

Urinary albumin excretion was measured by an immunoturbidemetric assay (Roche diagnostics, USA) on automated clinical chemistry analyzer (Hitachi 902). In all patients, the annual level was determined as the mean of urinary albumin excretions in three 24 h urine collections taken at home during normal physical activity. The intraassay and interassay coefficients of variation were 1.3 and 4.3 % for a mean value of 25 mg/l albumin concentration.

Serum glucose, cholesterol, triglyceride and HDL (Dade Behring, USA) were evaluated with enzymatic methods (Dade Behring, USA) on an automated clinical chemistry analyzer (Dimension RxL). Creatinin was assayed by the Jaffe reaction on an automated clinical chemistry analyzer (Dimension RxL).

HbA_1C _(reference range 4.4–6.0 %) was measured by a turbidimetric inhibition immunoassay technique (Roche Diagnostics, USA) on automated clinical chemistry analyzer (Hitachi 902). The intraassay and interassay coefficients of variation were 1.8 and 3.0 % for a mean value of 10.5 % HbA_1C _concentration.

The analysis of the data was performed with a PC compatible Instat-II programme. Paired t test and ANOVA were used where appropriate. Correlation analysis was performed with Spearman rank test. The differences were considered significant when the probability was p < 0.05. The results were given as mean ± SD.

## Results

The clinical characteristics of the study groups are shown in Table 1. There were no difference in age and BMI between the groups. Duration of diabetes mellitus was slightly longer, although not significantly different than that of the patients with proliferative retinopathy.

Results of laboratory parameters are shown in Table 2. Patients with proliferative retinopathy had higher concentrations of triglyceride and lower concentrations of HDL cholesterol as compared to diabetics without evidence of retinopathy and control group. Serum cholesterol level was the same in all study groups. Blood glucose and HbA_1C _levels were not different in subgroups of our diabetic population.

**Figure 1:**
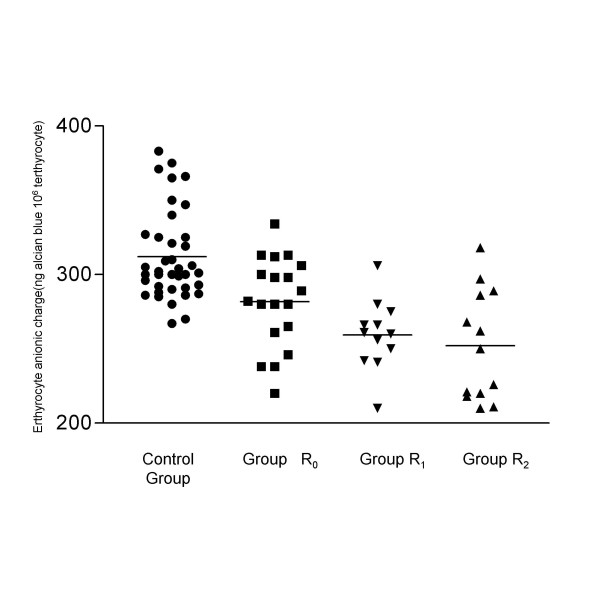
Distribution of erythrocyte anionic charges of all study groups.

Distribution of erythrocyte anionic charges of all study groups are demonstrated in figure 1. EAC was 312 ± 30 ng alcian blue/10^6 ^RBC in healthy controls. Diabetic patients both with nonproliferative and proliferative retinopathy had a significantly lower alcian blue binding to RBCs compared with diabetic patients without retinopathy (p < 0.001).

A marked (p < 0.05) difference was found between type 2 diabetic patients (8.3 ± 4.1, 8.7 ± 4.8, 8.4 ± 3.3 mg/24 h, Table 2) and controls (5.0 ± 2.4 mg/24 h) regarding urinary GAG excretion. We did not observe a correlation between urinary GAG excretion and diabetic retinopathy.

A significant difference (p < 0.05) was observed in serum sialic acid values in diabetic patients compared with healthy controls. Serum sialic acid level was higher in the nonproliferative retinopathy group, but the difference was statistically insignificant (p > 0.05).

Microalbumin level was significantly p < 0.05 higher in diabetic patients (73 ± 40 [35–250], 75 ± 21 [50–202], 85 ± 45 [56–224] mg/24 h, Table 2) compared to healthy controls (10 ± 6 mg/24 h). But there wasn't a correlation between the severity of retinopathy and microalbuminuria (p > 0.05).

Results of the Spearman rank correlation test are shown on table 3. EAC is found to be negatively corralated with microalbuminuria in all groups.

## Discussion

Our study demonstrates a statistically significant quantitative reduction of alcian blue binding to RBCs in diabetic patients than healthy subjects, which is in accordance with literature [15,16]. Previously we have shown a statistically significant quantitative reduction of alcian blue binding to RBCs in streptozosin diabetic rats [17]. Alcian blue is a complex amphoteric molecule that binds to acidic glycoproteins which represent a large class of cell surface anionic molecules [18]. As alcian blue binding is an expression of the anionic charge on the cell surface, this result indicates that RBC anionic charge is reduced in diabetic patients.

Erythrocyte anionic charge by itself is unlikely to be important in the pathogenesis of diabetic retinopathy but reduced RBC alcian blue binding was found to be associated with the loss of glomerular basement membrane anionic charges in diabetic rats and patients [19]. Changes in the composition of basement membranes are likely to be responsible for functional disturbances and hence for the development of capillary disease. Chakrabarti et al. reported a decreased density of anionic sites in the retinal basement membrane of BB rats [20]. A similar decrease in anionic density was also demonstrated in the Bruch's membrane of BB and streptozosin diabetic rats [21].

Several authors have questioned the validity of the alcian blue binding assay. The major objections concern the incomplete alcian blue dissolution in PBS buffer and its tendency to precipitate with time [22,23]. We prepared a fresh alcian blue solution for each experiment. Each experiment was considered valid when no change in the optical density of the blank alcian blue solution occurred over the duration of the experiment. In addition, the experiments were done in batches with cells from a healthy person. Therefore any flaw in the methodology would not affect only one group and thus distort the results.

Diabetic retinopathy is characterized by gradually progressive alterations in the retinal microvasculature leading to vascular hyperpermeability, capillary occlusion and ultimately neovascularization. Thickening of the retinal microvascular basement membrane and loss of anionic content are well documented morphological features of diabetic retinopathy [20,21,24]. These changes causes breakdown of the blood-retinal barrier resulting in capillary hyperpermeability and leakage of proteins into the deep and superficial layers of the retina [1,2].

Glycosaminoglycan primarily heparan sulfate has been implicated in permeability properties of basement membrane of glomerular basement membrane as well as the retinal basement membrane [25]. Kahaly et al. reported that urinary GAG excretion is increased in diabetic patients with microangiopathy [26]. Williamson et al and Ginn et al. have shown that albumin permeation is increased into the diabetic compared to normal retina [27,28]. Loss of heparan sulfate from the retinal basement membrane in addition to its thickening may cause increased vascular permeation of albumin and other substances into the retina and possibly into the optic nerve.

Systemic treatment of IDDM patients with proteinuria with danaparoid sodium, a glycosaminoglycan, not only reduced proteinuria, but also decreased the number of hard exudates in the retina [29]. Studies in diabetic rats have shown reduced activity of the key enzyme in the biosynthesis of heparan sulfate, N-deacetylase, which results in impaired heparan sulfate biosynthesis in experimental diabetes [30,31].

In this study we found that urinary GAG excretion is increased in diabetic patients than healthy controls, but there was no difference in GAG excretion between nonproliferative and proliferative retinopathy patients. We couldn't find a correlation between GAG excretion and EAC but we found a correlation between albumin excretion and EAC. Although our working hypothesis is that the reduction in anionic charge in basement membranes may be due to an abnormal GAG metabolism, we couldn't confirm it with this study.

Our study did not directly address the pathophysiology of diabetic retinopathy but a reduction in erythrocyte charge perhaps connected with the properties of retinal basement membrane.

Several reports suggest that elevated serum sialic acid levels in type 1 and type 2 diabetic patients indicate microvascular damage [32,33]. In our study, diabetic patients were found to have elevated serum sialic acid levels in comparison with healthy controls. Therefore, the increase in serum sialic acid level may indicate a coincidence between the structural alterations in the retina and general vascular damage.

## Conclusions

We conclude that type 2 diabetic patients with low erythrocyte anionic charge are associated with diabetic retinopathy. Reduction of negative charge of basement membranes may indicate general changes in microvasculature rather than retinopathy. More prospective and large studies needs to clarify the role of glycosaminoglycans on progression of retinopathy in type 2 diabetic patients.

## List of abbrevations

BMI: Body mass index

EAC: Erythrocyte anionic charge

GAG: Glycosaminoglycan

PBS: Phosphate buffered saline

RBC: Erythrocytes

U_GAG _: Urinary glycosaminoglycan

## Competing interests

The authors declare that they have no competing interests.

## Authors' contributions

**YB : **Evaluated laboratory parameters. Designed the study. Wrote the paper.

**HD: **Diagnosed outpatients according to American Diabetes Association criteria.

**MA: **Examined ocular fundus after dilation of the pupils and classified according to the severity of retinopathy.

**DY: **Performed the analysis of the data with a PC compatible Instat-II programme. Improved the manuscript.

## Pre-publication history

The pre-publication history for this paper can be accessed here:



## Figures and Tables

**Table 1:** Demographic characteristics of the study group

	Control group	Group R_0_	GroupR_1_	Group R_2_
Age(yrs)	57 ± 5	59 ± 8	56 ± 5	57 ± 5
M/F	19/19	13/10	7/6	7/6
BMI(kg/m^2 ^)	28 ± 5	27.8 ± 6	29.6 ± 5	28.5 ± 8
OAD/insulin	-	22/1	12/1	12/1
Duration of diabetes (yrs)	-	10 ± 5	11 ± 6	12 ± 8

**Table 2:** Laboratory parameters of the study groups

	Control group	Group R_0_	GroupR_1_	Group R_2_
Fasting glucose (mg/dl)	100 ± 26	229 ± 70	234 ± 89	236 ± 120
HbA_1c _(%)		7.6 ± 0.7	8.0 ± 0.7	8.0 ± 0.8
Cholesterol (mg/dl)	192 ± 44	187 ± 33	197 ± 41	193 ± 40
Triglycerides (mg/dl)	131 ± 55	153 ± 84	199 ± 71	228 ± 143
HDL (mg/dl)	45 ± 9	39 ± 9	37 ± 7	36 ± 10
Serum sialic acid (mmol/l)	1.81 ± 0.3	2.0 ± 0.5	2.1 ± 0.3	2.3 ± 0.5
EAC (ng alcian blue 10^6 ^erythrocyte)	312 ± 30	281 ± 30	259 ± 23	252 ± 37
U_GAG _(mg/24 h)	5.0 ± 2.4	8.3 ± 4.1	8.7 ± 4.8	8.4 ± 3.3
Microalbumin (mg/24 h)	10 ± 6	73 ± 40	75 ± 21	85 ± 45

**Table 3:** Correlation analysis between erythrocyte anionic charge (EAC) and other parameters.

	EAC (ng AB/ 10^6 ^erythrocyte)	
	r	P
HbA_1c _(%)	-0.251	0.073
Microalbumin (mg/24 h)	-0.393	0.032*
U_GAG _(mg/24 h)	-0.226	0.094
Serum sialic acid (mmol/L)	0.095	0.546
Duration of diabetes (yrs)	0.097	0.478

* Statistically significant at 0.05 level.
